# Severe Fever with Thrombocytopenia Syndrome Virus RNA in Semen, Japan

**DOI:** 10.3201/eid2511.190061

**Published:** 2019-11

**Authors:** Satoru Koga, Takahiro Takazono, Tsuyoshi Ando, Daisuke Hayasaka, Masato Tashiro, Tomomi Saijo, Shintaro Kurihara, Motohiro Sekino, Kazuko Yamamoto, Yoshifumi Imamura, Taiga Miyazaki, Katsunori Yanagihara, Kouichi Morita, Koichi Izumikawa, Hiroshi Mukae

**Affiliations:** Nagasaki University, Nagasaki, Japan

**Keywords:** severe fever with thrombocytopenia syndrome, semen, RNA virus, viruses, vector-borne infections, Japan, severe fever with thrombocytopenia syndrome virus, SFTS, SFTSV

## Abstract

Severe fever with thrombocytopenia syndrome virus (SFTSV) can be transmitted between humans. We describe a case of severe fever with thrombocytopenia syndrome in which SFTSV RNA was detected in semen after its disappearance from serum. Our findings indicate possible sexual transmission of this emerging virus.

Severe fever with thrombocytopenia syndrome (SFTS) is a life-threatening emerging infectious disease caused by severe fever with thrombocytopenia syndrome virus (SFTSV), a tickborne virus (genus *Banyang virus*, family *Phenuiviridae*). Recently, the person-to-person transmission of SFTSV has been described (*1*,*2*), and the most common risk factor of the transmission is direct blood exposure (*2*). However, SFTSV RNA has been detected in nonblood samples, such as throat, urine, and fecal specimens, especially in fatal cases (*3*). Asymptomatic infections through personal contact without blood exposure have also been reported (*1*). We describe a case in which viral RNA was detected in semen after viral RNA clearance from blood.

During May 2018, a previously healthy 50-year-old man hunted boar in the Goto Islands in western Japan. Eight days after hunting, he experienced high fever, myalgia, and diarrhea. He did not have hematuria or bloody diarrhea. Disturbance of consciousness occurred 6 days after symptom onset; on that day, he visited a local hospital and was referred to and admitted to Nagasaki University Hospital (Nagasaki, Japan). Body temperature was 39.0°C, and he was disoriented; Glasgow coma scale score was 9. He had no jaundice, signs of meningeal irritation, or apparent tick bites. Laboratory tests at admission had the following results: leukocytes 2.4 × 10^3^ cells/μL; platelets 35 × 10^3^/μL; serum creatine 3.04 mg/dL; aspartate aminotransferase 508 U/L; lactate dehydrogenase 1,404 U/L; and creatine kinase 15,449 U/L.

Because of the patient’s low platelet count and other suggestive signs and symptoms, we suspected SFTS. Serum SFTSV RNA level was 2.03 × 10^8^ copies/mL by real-time reverse transcription PCR (RT-PCR) analysis (Appendix). We confirmed diagnosis of SFTS on the basis of these results; however, we did not detect viral RNA in a urine sample. We conducted RT-PCR tests of semen and urine using procedures developed for serum; all RT-PCR tests were performed in the Department of Virology, Institute of Tropical Medicine, Nagasaki University, Nagasaki.

We considered this case severe, with multiple poor prognosis factors, such as disturbance of consciousness, laboratory data, and high viral load in serum (*4*). We performed palliative therapy, including continuous hemodiafiltration, mechanical ventilation, and central venous nutrition. In addition, we treated the patient with recombinant human soluble thrombomodulin for disseminated intravascular coagulation (380 U/kg/d for 6 d) and granulocyte colony-stimulating factor (filgrastim) for neutropenia (300 μg on the third hospital day). We also administered intravenous immunoglobulin (5,000 mg/d for 3 d), because it has been reported effective for SFTS (*5*), and the patient received platelet transfusions for severe thrombocytopenia.

We observed restoration of platelet count 10 days after symptom onset. Other abnormal laboratory findings recovered 7–13 days after symptom onset. The viral load in serum began to decrease from day 8 after onset and became negative on day 30 after onset. Although the patient’s general status was gradually improved and laboratory tests recovered to almost normal levels by day 30, we detected SFTSV RNA at 2.4 × 10^5^ copies/mL in his semen that day. On day 44, we could no longer detect semen SFTSV RNA, and he was discharged on day 51 after onset (Figure). 

**Figure:**
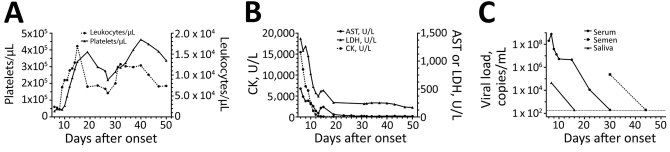
Laboratory data and viral loads during course of illness for patient with severe fever with thrombocytopenia syndrome, Japan. A) Leukocyte and platelet counts; B) AST, LDH, and CK levels; C) viral loads in serum, semen, and saliva. Dashed line in panel C indicates detection threshold (2 x 10^2^ copies/mL). AST, aspartate aminotransferase; CK, creatine kinase; LDH, lactate dehydrogenase.

In this study, SFTSV RNA was detected in semen, and SFTSV persisted longer in semen than in serum. It is well known that some viruses, such as Zika virus and Ebola virus, can be sexually transmitted; these viruses have been detected in semen for a prolonged period after symptom onset (*6*,*7*). Thus, we considered the potential risk for sexual transmission of SFTSV.

Compared with that of Zika and Ebola viruses, the clinical significance of potential sexual transmission of SFTSV is unknown. However, this possibility should be taken into consideration in sexually active patients with SFTSV. Our findings suggest the need for further studies of the genital fluid of SFTS patients, women as well as men, and counseling regarding sexual behavior for these patients.

Appendix Additional information about a case of severe fever with thrombocytopenia syndrome virus detected in semen, Japan.
