# Supplementary material for: Severe Fever with Thrombocytopenia Syndrome Virus RNA in Semen, Japan
Source: Emerg Infect Dis. 2019 Nov;25(11):2127–8. doi: 10.3201/eid2511.190061 (PMC6810197; doi:10.3201/eid2511.190061)
Supplement: Appendix — Additional information about a case of severe fever with thrombocytopenia syndrome virus detected in semen, Japan. [file 19-0061-Techapp-s1.pdf]

Article DOI: https://doi.org/10.3201/eid2511.190061

Severe Fever with Thrombocytopenia Syndrome Virus RNA in Semen, Japan

Appendix

RNA Extraction

We completed the following for serum, saliva, and semen samples.

For each sample, we mixed a 250 µL volume with 750 µL of Isogen-LS (Nippon Gene, https://www.nippongene.com) and extracted RNA according to the manufacturer's protocol. To efficiently extract RNA, we used Ethachinmate (Nippon Gene) as a co-precipitating agent in RNA precipitation with isopropanol. We dissolved the extracted RNA with 50 µL of RNase-free water and stored at −80°C until use.

Real-time RT-PCR

We measured Viral copy numbers by real-time RT-PCR as previously described (*1*). We designed SFTSV-specific primers and a probe based on the RdRp region. The sequence of forward (SFTS_QPCR_965F) primer was 5′-GCRAGGAGCAACAARCAAACATC-3′ and of reverse primer (SFTS_QPCR_1069R) was 5′-GCCTGAGTCGGTCTTGATGTC-3′. The PrimeTime qPCR probe was FAM/5′-CTCCCRCCC-3′/ZEN/5′-TGGCTACCAAAGC-3′/IBFQ (Integrated DNA Technologies, https://www.idtdna.com). The RT-PCR reaction was performed using a One Step PrimeScript RT-PCR Kit (Takara Bio Inc., www.takara-bio.com) and a 7500 Real-time RT-PCR System (Applied Biosystems, https://www.thermofisher.com). We quantified

the copy numbers by a standard curve method. We prepared the standard RNA as previously described (*2*), and adjusted concentrations at 10^7, 10^6, and 10^5 copies/5 μL. We used 5 μL of the standard RNase-free water as negative control and extracted RNA samples for the quantification. We performed all measurements in duplicate.

References

1. Hayasaka D, Shimada S, Aoki K, Takamatsu Y, Uchida L, Horio M, et al. Epidemiological survey of severe fever with thrombocytopenia syndrome virus in ticks in Nagasaki, Japan. Trop Med Health. 2015;43:159–64. PubMed https://doi.org/10.2149/tmh.2015-01

2. Hayasaka D, Aoki K, Morita K. Development of simple and rapid assay to detect viral RNA of tick-borne encephalitis virus by reverse transcription-loop-mediated isothermal amplification. Virol J. 2013;10:68. PubMed https://doi.org/10.1186/1743-422X-10-68
